# Small Molecule RPI-194 Stabilizes Activated Troponin to Increase the Calcium Sensitivity of Striated Muscle Contraction

**DOI:** 10.3389/fphys.2022.892979

**Published:** 2022-06-08

**Authors:** Zabed Mahmud, Svetlana Tikunova, Natalya Belevych, Cory S. Wagg, Pavel Zhabyeyev, Philip B. Liu, David V. Rasicci, Christopher M. Yengo, Gavin Y. Oudit, Gary D. Lopaschuk, Peter J. Reiser, Jonathan P. Davis, Peter M. Hwang

**Affiliations:** ^1^ Department of Biochemistry, University of Alberta, Edmonton, AB, Canada; ^2^ Department of Physiology and Cell Biology, The Ohio State University, Columbus, OH, United States; ^3^ Division of Biosciences, College of Dentistry, The Ohio State University, Columbus, OH, United States; ^4^ Department of Pediatrics, University of Alberta, Edmonton, AB, Canada; ^5^ Department of Medicine, University of Alberta, Edmonton, AB, Canada; ^6^ Department of Cellular and Molecular Physiology, College of Medicine, Pennsylvania State University, University Park, PA, United States

**Keywords:** cardiac troponin activator, calcium sensitizer, inotrope, systolic heart failure, striated muscle, thin filament

## Abstract

Small molecule cardiac troponin activators could potentially enhance cardiac muscle contraction in the treatment of systolic heart failure. We designed a small molecule, RPI-194, to bind cardiac/slow skeletal muscle troponin (Cardiac muscle and slow skeletal muscle share a common isoform of the troponin C subunit.) Using solution NMR and stopped flow fluorescence spectroscopy, we determined that RPI-194 binds to cardiac troponin with a dissociation constant K_D_ of 6–24 μM, stabilizing the activated complex between troponin C and the switch region of troponin I. The interaction between RPI-194 and troponin C is weak (K_D_ 311 μM) in the absence of the switch region. RPI-194 acts as a calcium sensitizer, shifting the pCa_50_ of isometric contraction from 6.28 to 6.99 in mouse slow skeletal muscle fibers and from 5.68 to 5.96 in skinned cardiac trabeculae at 100 μM concentration. There is also some cross-reactivity with fast skeletal muscle fibers (pCa_50_ increases from 6.27 to 6.52). In the slack test performed on the same skinned skeletal muscle fibers, RPI-194 slowed the velocity of unloaded shortening at saturating calcium concentrations, suggesting that it slows the rate of actin-myosin cross-bridge cycling under these conditions. However, RPI-194 had no effect on the ATPase activity of purified actin-myosin. In isolated unloaded mouse cardiomyocytes, RPI-194 markedly decreased the velocity and amplitude of contractions. In contrast, cardiac function was preserved in mouse isolated perfused working hearts. In summary, the novel troponin activator RPI-194 acts as a calcium sensitizer in all striated muscle types. Surprisingly, it also slows the velocity of unloaded contraction, but the cause and significance of this is uncertain at this time. RPI-194 represents a new class of non-specific troponin activator that could potentially be used either to enhance cardiac muscle contractility in the setting of systolic heart failure or to enhance skeletal muscle contraction in neuromuscular disorders.

## Introduction

Heart failure is a common disease condition in which the heart is unable to pump enough blood to satisfy the metabolic demands of the body. Systolic heart failure, also known as heart failure with reduced ejection fraction (HFrEF), occurs when inadequate contraction results in an ejection fraction of less than 40% (Murphy et al., 2020). Over time, the heart becomes increasingly thinned and dilated, which further exacerbates muscle wall tension to impair contraction. Atherosclerotic ischemic heart disease is the most common etiology causing HFrEF (Curtis et al., 2003). In decompensated heart failure, blood pressure is often low, the perfusion of vital organs is barely adequate, and fluid accumulates throughout the body due to maladaptive sodium retention by the kidneys. The most well-established drug therapies in heart failure are diuretics to reverse volume overload and blood pressure medications that attenuate long term pathologic remodeling of the heart. What is missing from the therapeutic arsenal is an effective positive inotrope, a drug that increases the contractility of the heart, because to date no existing positive inotrope has been shown to improve survival.

The oldest therapy for heart failure, digoxin, inhibits cellular Na, K-ATPase function and increases cardiac muscle contraction through an increase in cytoplasmic calcium concentration. Digoxin therapy improves symptoms and reduces hospitalization rates for heart failure (Bourge et al., 2013), but a narrow therapeutic index has limited its use so that it is no longer a recommended therapy. The most commonly used positive inotropes in the intensive care unit, β_1_-agonists like dobutamine and downstream type 3/4-phosphodiesterase (PDE3/PDE4) inhibitors like milrinone increase cardiac output, but they also confer a risk of tachyarrhythmias and promote peripheral vasodilation and hypotension. Due to adverse side effects, these agents do not provide a survival benefit in chronic or acute decompensated heart failure (Tacon et al., 2012).

In theory, directly targeting the sarcomeric proteins that generate cardiac muscle contraction could enhance cardiac output with fewer side effects (Kitada et al., 1989; Wolska et al., 1996; Brixius et al., 2000; Li et al., 2000; Tikunova et al., 2010; Hwang and Sykes, 2015; Shettigar et al., 2016). Omecamtiv mecarbil is a compound that binds to cardiac myosin to stabilize its pre-powerstroke conformation (Planelles-Herrero et al., 2017). This increases the number of strong actin-myosin cross-bridges, enhancing cooperative activation of the cardiac thin filament. Though omecamtiv enhances actin-myosin interactions, it was also found to suppress the myosin working stroke (Woody et al., 2018) and to decrease actin sliding velocity in *in vitro* motility assay (Swenson et al., 2017), giving it a mixed activation/inhibition mechanism of action. Omecamtiv mecarbil prolongs the systolic phase of the cardiac cycle and increases the ejection fraction of the left ventricle, though it does not enhance the speed or force of contraction (Malik et al., 2011). Phase III clinical trials of omecamtiv mecarbil have not shown a statistically significant survival benefit in chronic (Teerlink et al., 2021) or acute decompensated heart failure (Teerlink et al., 2016).

It may be more advantageous to enhance activation of the thin filament without modulating the force-generating ATPase cycle of myosin (Shettigar et al., 2016). Thin filaments in cardiac sarcomeres are activated by cardiac troponin (cTn) (Li et al., 2004; Metzger and Westfall, 2004; Shettigar et al., 2016), which consists of three protein subunits: calcium-binding cTnC, actin binding inhibitory cTnI, and tropomyosin binding cTnT (Ebashi and Ebashi, 1964; Ebashi et al., 1967; Greaser and Gergely, 1973). X-ray crystallography (Takeda et al., 2003) and NMR (Sia et al., 1997) studies revealed that the cTnC subunit is a dumbbell-shaped protein with two globular domains, the N-terminal regulatory domain (cNTnC) and the C-terminal structural domain (cCTnC). The cNTnC domain has two EF-hand motifs, EF-I and EF-II, but only EF-II is active and binds calcium with micromolar affinity, attuned to sense the increase in free cytoplasmic calcium concentration during systole (the contractile phase of the cardiac cycle). Calcium ions come on and off cNTnC very rapidly (rate of exchange, k_ex_ > 5,000 s^−1^) (Li et al., 2002), with the calcium-bound state experiencing a rapid equilibrium between closed and partially open conformations (Sia et al., 1997; Spyracopoulos et al., 1997). Binding of the switch region of cTnI (cTnI_148-158_) to cNTnC stabilizes its calcium-bound open state, substantially slowing calcium dissociation (Li et al., 2002; Siddiqui et al., 2016). Cryo-EM structures of cTn (Oda et al., 2020; Yamada et al., 2020) showed that residues 135–209 of cTnI bind to actin to maintain the thin filament in a blocked state, but binding of cNTnC to cTnI_148-158_ relieves the inhibition of the thin filament, shifting tropomyosin from its blocked position and facilitating strong actin-myosin interaction.

Troponin exists in three different isoforms found in fast skeletal, slow skeletal, and cardiac muscle. For troponin I and troponin T, there are three different isoforms which are specific for each muscle type, but cardiac muscle and slow skeletal muscle share the same isoform of for troponin C (*i.e.*, cTnC = ssTnC) (Li and Hwang, 2015). The fast skeletal isoform of TnC (fsTnC) has been specifically targeted by the drugs tirasemtiv and reldesemtiv, which completed clinical trials for the treatment of amyotrophic lateral sclerosis, though benefit was limited (Shefner et al., 2019; Shefner et al., 2021). Reldesemtiv is currently undergoing clinical trials for the treatment of spinal muscular atrophy (Rudnicki et al., 2021). These fsTnC-targeting drugs bind to a hydrophobic cavity in fsTnC that lies beneath the binding site for the fsTnI switch region (Li et al., 2021). In theory, it should be possible to develop a compound that targets the homologous binding cavity in cTnC/ssTnC, though it would likely be active for both cardiac and slow skeletal muscle. Cytokinetics has developed a cardio-selective troponin activator, CK-136, formerly known as AMG 594, which appears to be selective for cardiac muscle, though a structure of its binding site on cardiac troponin is not yet available (He et al., 2021).

Previous attempts to design positive inotropes appeared to target cardiac troponin but in fact resulted in compounds that also bind to other targets in cardiomyocytes. Such compounds include levosimendan, pimobendan, MCI-154, and EMD 57033. Of these, levosimendan (Orstavik et al., 2015), pimobendan (Bohm et al., 1991), and MCI-154 (Bethke et al., 1993; Li et al., 2018) were found to have potent PDE3-inhibitory activity, whereas EMD 57033 was found to interact with cardiac myosin (Brixius et al., 2000). These compounds have lower affinity for cTnC than for these other proteins.

We, therefore, designed and screened compounds for binding to the cTn complex using a unique cNTnC-cTnI chimeric construct, which we named “gChimera” (Cai et al., 2018). We synthesized a novel small molecule cardiac troponin modulator, RPI-194, and measured its binding to both gChimera and to the isolated cNTnC domain, as well as its activity in skinned cardiac muscle trabeculae, individual cardiomyocytes, and isolated perfused working mouse hearts. Since cardiac muscle shares the same TnC isoform as slow skeletal muscle, we have also examined its activity in skinned skeletal muscle fibers. Slow skeletal muscle has a distinct isoform of troponin I [ssTnI], but the switch region of ssTnI that binds cNTnC is very similar to the corresponding region in cTnI. Our goal was to develop a compound that can be used as a positive inotropic agent in the treatment of systolic heart failure, but it turns out that our compound interacts with troponin from all striated muscle types.

## Materials and Methods

### Preparation of Proteins for NMR Studies

Three human protein constructs were used in the NMR study: 1) recombinant human aCys-cNTnC (C35S, C84S double mutant), 2) chimeric construct (gChimera) of the cNTnC-cTnI switch peptide complex, aCys-cNTnC_1-85_—SSGGSSGGSSGG linker - cTnI_145-167_ and 3) slow skeletal troponin I switch peptide (ssTnI). The protocol used to express and purify both cNTnC and gChimera in *Escherichia coli* was previously described (Cai et al., 2016). The ssTnI peptide was synthesized and purified by GL Biochem Ltd. (Shanghai).

### NMR Titration of RPI-194 Against gChimera and cNTnC

RPI-194 was synthesized by Rane Pharmaceuticals, Inc. in Edmonton, Alberta, Canada. Chemical structure was confirmed by NMR. For each NMR titration experiment, recombinant ^15^N-labeled gChimera or cNTnC was dissolved in 500 µL NMR buffer (90% H_2_O/10% D_2_O) consisting of 100 mM KCl, 10 mM imidazole, and 0.5 mM 4, 4-dimethyl-4-silapentane-1-sulfonic acid as a chemical shift reference. Purified lyophilized forms of gChimera or cNTnC were dissolved in NMR buffer. Protein quantitation by acid hydrolysis followed by amino acid quantitation showed the lyophilized form was 54% pure protein by weight. The pH of each NMR sample was maintained at a slightly acidic pH ∼ 6.7 by adjusting with microliter quantities of either 1 M NaOH or 1 M HCl. An acidic pH is typically employed in NMR to slow down base-catalyzed solvent-amide exchange, improving the signal intensity for rapidly exchanging amide groups in the protein. Since the proteins of interest are not known to have native side chains that become ionized near pH 6.7 (typically histidine), the use of a slightly acidic pH should not impact the electrostatic surface of the protein at all.

RPI-194 compound was dissolved into d_6_-DMSO to make a 68 mM stock solution, which was then diluted ten-fold in d_6_-DMSO to perform titrations. For both cNTnC and gChimera, the starting concentration was 115 µM. RPI-194 was titrated to 0.1, 0.2, 0.3, 0.4, 0.6, 0.8, 1, 1.5, 2, 2.5, 3, 3.5, 4, 5, 6 and 8 equivalents of cNTnC. For gChimera-RPI-194 titration, RPI-194 was titrated to 0.2, 0.4, 0.6, 0.8, 1, 1.2 and 1.4 equivalents of gChimera. Each titration point was monitored by recording a two dimensional ^1^H, ^15^N heteronuclear single quantum coherence (HSQC) spectrum. Dilution factors were applied at each titration point to calculate the final concentration of cNTnC and RPI-194 in the calculation of binding affinities.

### NMR Titration of ssTnI Against cNTnC and cNTnC·RPI-194 Complex

Titration of ssTnI was performed against free cNTnC and against cNTnC complexed with RPI-194. A 10 mM stock concentration of ssTnI was made by dissolving it into d_6_-DMSO. ssTnI was titrated to 0.5, 1, 1.5, 2, 2.5, 3, 3.5, 4, 5, 6, 8, 10, 13, 16, 20, 25 and 30 equivalents of cNTnC. For titrating ssTnI into cNTnC·RPI-194 complex, RPI-194 was first titrated into free cTnC (115 µM) until both protein and drug were 1:1 equivalent. Then ssTnI was titrated with 0.1, 0.2, 0.4, 0.6, 0.8, 1, 1.5, 2, 2.5, and 3 equivalents of cNTnC·RPI-194 complex (115 µM).

### NMR Spectroscopy

All titrations were performed on a Varian Inova 500 MHz NMR spectrometer equipped with triple resonance ^1^H, ^13^C, ^15^N probe. 2D ^1^H, ^15^N HSQC spectra were collected for each titration point at 30°C. All titration data were processed using NMRPipe (Delaglio et al., 1995). A MATLAB runtime-based two dimensional lineshape analysis program called TITAN was used to calculate the dissociation constant (K_D_) from titration experiments (Waudby et al., 2016). First, the protein and ligand concentration of each titration point were specified in the program. Individual NMR spectra for each titration point were uploaded into TITAN, and a specific region of interest (ROI) was selected for peaks we selected for large chemical shift perturbations. TITAN only considers chemical shift changes within the selected ROI for fitting and calculating dissociation constants. Simplified two-state binding models were used to calculate binding dissociation constants and on/off rate constants.

### Determination of Binding Affinities by Steady State Fluorescence

All steady-state fluorescence measurements were obtained on a SpectraMax i3x multi-mode microplate reader at 15°C. RPI-194 has intrinsic fluorescence with peak excitation and emission wavelengths at 335 and 470 nm, respectively. The same cNTnC and gChimera proteins (0–297 µM) used for NMR were titrated into a solution containing 5 µM RPI-194, 50 mM HEPES, 150 mM KCl, 5 mM MgCl_2_, 1 mM DTT and 10 mM CaCl_2_, and the intrinsic fluorescence spectrum of RPI-194 was monitored for changes. Binding affinities were calculated using GraphPad Prism version 9.0.2 (San Diego, California, United States).

### Determination of Ca^2+^ Dissociation Rates by Stopped-Flow Fluorescence

Recombinant human cTnC (T53C, C35S, and C84S), cTnI and cTnT were used to reconstitute the cardiac troponin complex for stopped flow fluorescence studies. Expression, purification, production, and labeling of cTnC T53C with 2-(4′-(iodoacetamido) anilino) naphthalene-6-sulfonic acid (IAANS) were previously published (Davis et al., 2007). Expression and purification of recombinant cTnI, cTnT and reconstitution of the cardiac troponin complex (cTnC cTnI·cTnT) were as previously described (Davis et al., 2007).

Calcium release rates of IAANS-labeled, reconstituted cardiac troponin complex as a function of RPI-194 concentration were measured in a stopped-flow spectrometer (Applied Photophysics model SX.18 MV). IAANS excitation and emission were monitored at 330 nm and 420–470 nm, respectively. The calcium release rate was monitored by mixing calcium saturated (500 μM Ca^2+^) cardiac troponin complex with a stopped flow buffer containing calcium chelating solution (EGTA 10 mM, 10 mM MOPS and 150 mM KCl, pH 7.0) with a dead mixing time ∼ 1.24 ms. EGTA (10 mM) was used to sequester calcium from reconstituted cardiac troponin complex (0.3 µM) in the absence or presence of RPI-194. Increasing concentrations of RPI-194 were used in this reaction. P.J. King data analysis software, developed by Applied Photophysics (Leatherhead, Surrey, UK), was used to analyze stopped flow data. It uses a nonlinear Levenberg–Marquardt algorithm for data fitting.

### Analysis of Force *Versus* pCa Relationship in Skinned Ventricular Trabeculae and Skeletal Muscle Fibers

Heart, soleus, and tibialis anterior muscles were isolated from each of 8 male Sprague-Dawley rats, ranging in age from 6–9 months. The rats were euthanized (anesthesia induced by isoflurane, followed by rapid cardiectomy) in accordance with a protocol approved by Institutional Animal Care and Use Committee of Ohio State University. The soleus and anterior tibialis muscles were immediately placed in cold relaxing solution, and fiber bundles were prepared and stored in relaxing solution containing 50% glycerol (v/v) at −20°C (Reiser et al., 2013). A single large cut was made through the free wall of both ventricles of the heart, which was then placed in ice-cold relaxing solution with 1% Triton X-100 for 30 min (Tikunova et al., 2019). The heart was removed from this solution, gently compressed, and blotted and transferred to cold glycerinating solution (Reiser et al., 2013).

Single trabeculae were isolated and studied as previously described (Tikunova et al., 2019). Briefly, a trabecula was mounted in the experimental chamber that was controlled at 15°C (Tikunova et al., 2019). In the chamber, one end of the trabecula was attached to a motor and another end was attached to a transducer. The trabecula was set to the resting striation spacing, the equivalent of sarcomere length. Striation spacing was determined using a camera mounted on the microscope and the SPOT image analysis software (https://www.spotimaging.com) (Tikunova et al., 2019). The distance spanned by ∼ 20 striations was measured to calculate resting sarcomere length. Fiber width and depth were measured, and fiber cross-sectional area (CSA) was calculated, assuming an ellipsoidal cross section. The average resting sarcomere length of the twenty-four trabeculae that were studied was set to 2.07 ± 0.02 μm. Each trabecula was then subjected to two series, A and B, of activations. Series A was always without RPI-194. Series B was with 0, 20, 50 or 100 µM RPI-194.

The force versus pCa relationship was measured in six trabeculae for each concentration of RPI-194, first without (series A), then with (series B) RPI-194 (100 mM stock dissolved in DMSO) (Tikunova et al., 2019). RPI-194 was added to all of the solutions to which the trabeculae were exposed during the measurements of the force/pCa relationship: pCa 9.0 solution, HDTA pre-activating solution and each of the maximal (pCa 4.0) and submaximal activating solutions. The trabeculae were soaked in pCa 9.0 solution with RPI-194 at 15°C for 30 min before initiating the second series of force measurements. We reported previously that DMSO had no effect on the force/pCa relationship (Reiser et al., 2013). We initially determined, in three skeletal muscle fibers, that the control (no added compound) force/pCa relationship is essentially identical when measured twice in a given preparation. The trabecula was treated with series of pCa solutions as previously described (Reiser et al., 2013). The force versus pCa data were fit as previously described (Black et al., 2000; Tikunova et al., 2002; Tikunova et al., 2019).

The sarcomere length in slow and fast fibers was measured using the Fast Fourier Transform in ImageJ (https://imagej.nih.gov/ij/). The fiber type (slow or fast) of each studied skeletal muscle fiber was determined from an analysis of the myosin heavy chain isoform composition using SDS-PAGE, as described (Tikunova et al., 2018). The maximal velocity of shortening (V_o_) was measured, using the slack test (Edman, 1979), in slow and fast fibers when activated in pCa 4.0 solution (every third activation in the force/pCa measurements series). Thus, two V_o_ determinations were made in each slow and fast fiber, first without, then with, RPI-194 (series A and B).

A total of 24 slow fibers, 24 fast fibers and 24 trabeculae were studied. The group size for each concentration of RPI-194 was six and each preparation was studied at one concentration. An analysis of variance (ANOVA) Tukey’s post-hoc test was used when comparing the effects of all four concentrations (0, 20, 50 and 100 µM) of RPI-194 in each preparation (slow fibers, fast fibers or trabeculae) or when comparing effects of a given concentration of RPI-194 in all three preparations (slow fibers, fast fibers and trabeculae). Student’s t-test was used to assess the significance of differences in maximal shortening velocity in fast and slow fibers at a given concentration of RPI-194. Pearson’s correlation was used to test for a relationship between the shift in pCa_50_ and the change in V_o_ induced by RPI-194.

### Isolation of Mouse Ventricular Myocytes, Contractility Assays and cAMP Measurements

Adult ventricular cardiomyocytes were isolated and perfused as previously described (Sah et al., 2002). Contractility assays from isolated cardiomyocytes were conducted as previously described (Crackower et al., 2002). Briefly, a Grass S44 stimulator with a pulse duration of 3 milliseconds at 1 Hz was used to stimulate cardiomyocytes. Myocyte contraction was tracked at 240 Hz using a video edge detector. In addition, myocyte steady state contractions at 1 Hz and a subsequent equilibrium period for 4 min at 240 Hz were recorded. We determined fractional shortening, shortening rate (+dL/dT) and relaxation rate (dl/dT) in the isolated cardiomyocytes.

### Isolated Mouse Working Heart Perfusion and Measurement of Metabolic Rates

All animals used in isolated working heart perfusion experiments were treated in accordance with the guidelines of the Canadian Council of Animal Care and approved by the University of Alberta Health Sciences Animal Welfare Committee. All animal experiments were conducted on male C57BL/6 mice (7–10 weeks) obtained from Charles River Laboratories (Wilmington, MA, United States) and regularly fed with chow diet (Harlan Teklad, Madison, WI, United States). Animals were anesthetized with 60 mg kg^−1^ isoflurane administered through the peritoneum. Isolated working heart perfusions were performed as previously described (Kuang et al., 2004). Rapidly excised hearts were immediately placed on an ice-cold Krebs-Henseleit solution. A recirculating perfusate solution was used for the isolation of working hearts. It consisted of a modified Krebs-Henseleit solution (100 ml) which was a mixture of 1.2 mM KH_2_PO_4_, 1.2 mM MgSO4, 2.5 mM CaCl_2_, 4.7 mM KCl, 25 mM NaHCO_3_ and 118 mM NaCl. The perfusate was supplemented with 1.2 mM palmitate prebound to 3% bovine serum albumin and 5 mM glucose as energy substrates. Glycolysis and glucose oxidation rates were calculated from the perfused heart by adding a small amount of radiolabeled [5-^3^H] glucose and [U-^14^C] glucose in the Krebs-Henseleit solution (Kovacic et al., 2003; Kuang et al., 2004). The perfusate was continuously supplied with a gas mixture of 95% O_2_, 5% CO_2_. Cardiac output, cardiac work, heart rate and peak systolic pressure were also assessed from the perfused hearts.

### Impact of RPI-194 on Cardiac Myosin ATPase Activity

The ATPase activity of human beta-cardiac myosin subfragment 1 (amino acids 1–843) containing a C-terminal green fluorescent protein tag (M2β-S1 GFP) was examined using the NADH coupled assay (Swenson et al., 2017; Tang et al., 2019). M2β-S1 GFP was produced using the C2C12 cell expression system and purified as previously described (Swenson et al., 2017; Tang et al., 2019). The ATPase activity was examined in the presence of 40 µM actin and varying RPI-194 concentrations with 1% DMSO present.

## Results

### RPI-194 Binds Ca^2+^ Saturated cNTnC-cTnI Chimera

**FIGURE 1:**
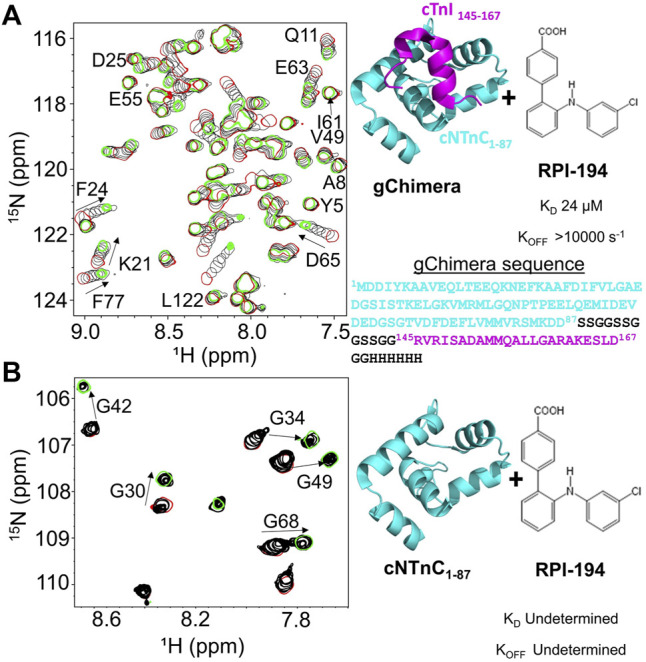
**(A)** Titration of RPI-194 compound into ^15^N-labeled gChimera tracked by 2D ^15^N-HSQC NMR spectra (left). Start and end points colored as red and green, respectively. Direction of chemical shift perturbation is marked with arrows. The gChimera structure with its amino acid sequence is shown on the right **(B)** RPI-194 titration into ^15^N-labeled cNTnC domain causes signal broadening and disappearance in some residues (left).

During systole, cardiac muscle contraction is triggered by the calcium dependent binding of the cTnI switch region to the regulatory cNTnC. A cardiac troponin activator drug promotes and stabilizes formation of this activated complex. We previously produced multiple variations of the cNTnC-cTnI chimera with different linkers (Pineda-Sanabria et al., 2014; Cai et al., 2016). In the current study we used a further refined version that we call “gChimera”, which utilizes a linker containing multiple Ser and Gly residues for maximum flexibility and solubility while maintaining charge neutrality (amino acid sequence shown in Figure 1A).

Based on our previous work, 3-chlorodiphenylamine was a promising starting compound to develop a cardiac troponin activator (Cai et al., 2016). Addition of hydrophobic substituents to the aryl rings of 3-chlorodiphenylamine tends to improve binding affinity but greatly reduces solubility, whereas more polar substituents are not well tolerated. We aimed to add at least one hydrophilic group to enhance solubility and specificity of binding. We designed a total of 54 3-chlorodiphenylamine-based compounds that were synthesized by Rane Pharmaceutical Inc., Edmonton, AB, Canada. The compounds were assessed for binding to gChimera by NMR, and we identified a compound, RPI-194, which has an additional *p*-benzoic acid in the ortho position of the aniline group, with a measured dissociation constant, K_D_, of 24 µM (see Figure 1A). Linear migration of the NMR signals suggests 1:1 binding kinetics in the fast exchange regime. (“Fast” exchange means fast relative to the frequency differences between NMR signals in the two different states.) We also calculated the binding affinity by titrating gChimera into RPI-194, monitoring the steady state intrinsic fluorescence of the RPI-194 compound to yield a measured dissociation constant, K_D,_ of 14 µM (see Supplementary Figure S1).

### RPI-194 Binds Weakly to Ca^2+^-Saturated cNTnC in the Absence of cTnI Switch Peptide

RPI-194 binds to the isolated cNTnC domain with a lower affinity than gChimera (K_D_ = 300 µM), as measured by steady state fluorescence (see Supplementary Figure S1). Compared with gChimera, the isolated cNTnC domain lacks the cTnI switch region, which shifts it to an open conformation and binds to small molecules like RPI-194 via the side chains of residues Ile148 and Met153.

NMR titration experiments show a complex equilibrium when RPI-194 is titrated into the cNTnC domain, precluding binding affinity determination by NMR, as was done for gChimera. Prior to addition of RPI-194, the NMR spectrum of isolated cNTnC domain demonstrates signal broadening due to fast timescale conformational exchange between closed and open states (Sia et al., 1997; Spyracopoulos et al., 1997), undergoing a closed-to-open transition with a k_ex_ of about 30,000 s^−1^ (Eichmuller and Skrynnikov, 2005), with the more open conformation representing a minor population of about 5% (Mckay et al., 2000; Paakkonen et al., 2000). Peaks shift and then rapidly disappear upon addition of RPI-194, indicating intermediate timescale binding, consistent with selective binding of RPI-194 to the less populated open state (see Figure 1B). As more RPI-194 is added, new NMR signals corresponding to RPI-194-cNTnC complex abruptly re-appear, but in some cases (for example, for residues G30 and G42) they appear in a different position than one would expect based on the start of the titration. This suggests a new conformational process occurring different from the initial 1:1 binding of cNTnC to RPI-194. Other peaks in the spectrum that do not shift become visibly reduced in intensity, suggesting a large increase in molecular weight consistent with dimerization.

Similar changes occur when the drug trifluoperazine is titrated into calmodulin, a protein homologous to troponin C (Feldkamp et al., 2010; Waudby et al., 2016). Each homologous domain of calmodulin binds two molecules of trifluoperazine, and this promotes association of the N-terminal domain with the C-terminal domain through hydrophobic interactions. We propose that at high concentrations of RPI-194, one or two molecules of RPI-194 bind and stabilize the open conformation of the cNTnC domain, which then has a tendency to dimerize. Physiologically, the cNTnC domain does not dimerize because cTnC is tethered to fixed positions along the thin filament. Moreover, the cNTnC domain is predominantly in the closed state unless the cTnI switch region is bound. Thus, while the behaviour of free cNTnC domain in the presence of RPI-194 (and many other compounds) is interesting in terms of its tendency to dimerize, it is not physiologically relevant, except to note that RPI-194 does bind to calcium-saturated cNTnC domain in the absence of cTnI switch region, though binding is substantially more effective once the cTnI switch region is bound. This suggests that RPI-194 is more effective at stabilizing the activated troponin complex once it is formed, rather than promoting the formation of the activated complex.

### RPI-194 Enhances Binding of ssTnI Switch Peptide to cNTnC

To examine the impact of RPI-194 on binding of TnI switch peptide to cNTnC, we titrated TnI switch peptide into isolated cNTnC domain. Cardiac muscle troponin C (cTnC) is the same isoform as slow skeletal muscle troponin C (ssTnC), though slow skeletal muscle possesses different isoforms of troponin I (ssTnI) and troponin T (ssTnT). We used the ssTnI switch peptide instead of the cardiac isoform because of its superior solubility. The cTnI switch peptide readily precipitates out of solution when titrated into solutions containing cTnC and RPI-194, making determination of binding constants unreliable.

**FIGURE 2:**
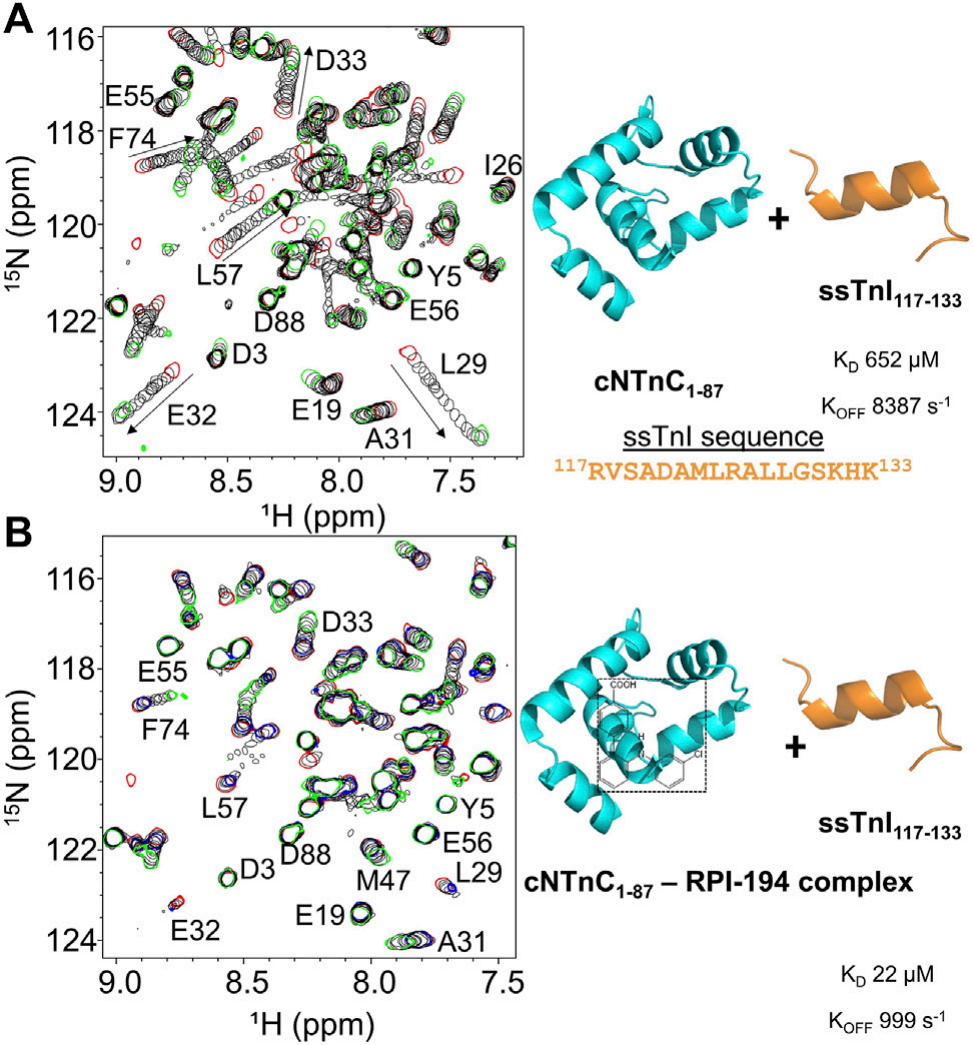
**(A)** 2D ^15^N-HSQC spectra of ssTnI titration into ^15^N-labeled cNTnC (left). Titration start and end points are colored as red and green, respectively. The direction of chemical shift perturbation is indicated with arrows **(B)** The 2D ^15^N-HSQC spectra of ssTnI titration into ^15^N-labeled cNTnC and unlabeled RPI-194 complex (left). cNTnC·RPI-194 titration start and end points are colored as red and blue, respectively. End of cNTnC·RPI-194·ssTnI titration is colored green.

When ssTnI switch peptide (as opposed to small molecule RPI-194) is titrated into cNTnC, signals that were broad at the start of the titration progressively become narrower as the switch peptide shifts the cNTnC conformational equilibrium to a fully open state (see Figure 2A), indicating fast kinetics of binding. This is in marked contrast to when RPI-194 is titrated into cNTnC, in which peaks that were broad at the beginning of the titration become broadened beyond detection as RPI-194 is added. Thus, the ssTnI switch peptide appears able to bind cNTnC via a rapid induced fit mechanism, whereby it stimulates the transition of cNTnC from a closed to an open state, whereas RPI-194 binds via conformational selection, requiring a stochastic transition to the open state prior to binding. Using a two-dimensional lineshape analysis tool, TITAN, we calculated the ssTnI switch peptide binding affinity for cNTnC (K_D_ 652 µM) that is weaker than that previously determined for the corresponding cTnI switch peptide (K_D_ 154 µM) (Li et al., 2002).

We then titrated ssTnI switch peptide into cNTnC domain in the presence of RPI-194 (Figure 2B). The presence of one equivalent of RPI-194 significantly enhances the binding of ssTnI switch peptide to cNTnC. The binding affinity of ssTnI switch peptide for the cNTnC: RPI-194 complex is K_D_ 22 µM as calculated by TITAN, which is significantly tighter than the value of 652 µM determined for cNTnC-ssTnI binding in the absence RPI-194, over an order of magnitude change. Improved binding of the TnI switch peptide is consistent with previous NMR studies of the cardiac troponin activator dfbp-o (Robertson et al., 2010; Lindert et al., 2015).

### RPI-194 Slows Calcium Release From the Trimeric Cardiac Troponin Complex

**FIGURE 3:**
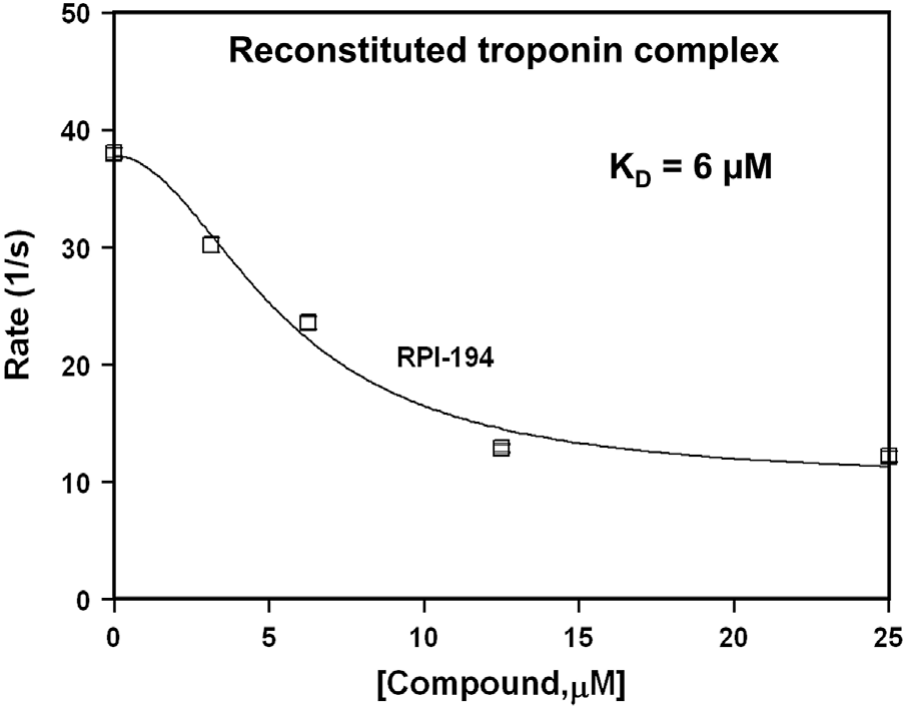
Stopped flow fluorescence experiments. Rate of calcium release from IAANS-labeled reconstituted troponin complex as a function of RPI-194 concentration. *N* = 10 for all measurements.

We used stopped-flow fluorescence of IAANS-labeled troponin C to measure the impact of RPI-194 on calcium release rates in troponin. RPI-194 binding to reconstituted heterotrimeric cardiac troponin complex slowed the rate of calcium release from 38 s^−1^ to 13 s^−1^ (Figure 3), with an apparent dissociation constant, K_D_, of 6 μM, in agreement with NMR and steady state fluorescence measurements. This is consistent with our NMR studies demonstrating that RPI-194 stabilizes the calcium-saturated activated troponin complex.

### RPI-194 Activates Cardiac, Slow Skeletal, and Fast Skeletal Muscle in Isometric Contraction, but Slows the Velocity of Unloaded Contraction in Skeletal Muscle

**FIGURE 4:**
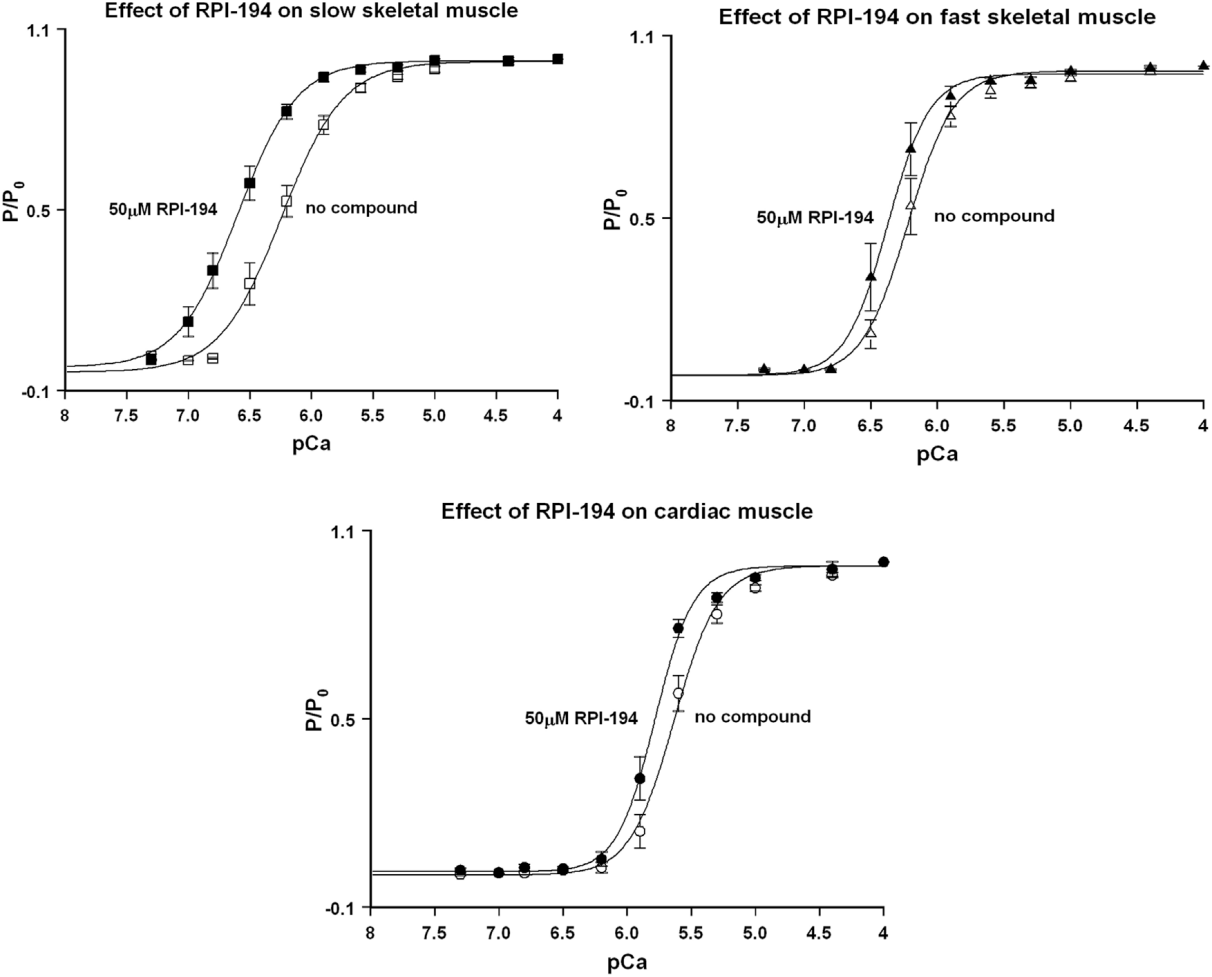
Normalized force (P/P_o_) versus pCa curves for rat skinned cardiac trabeculae, fast skeletal, and slow skeletal muscle fibers in the presence of 50 µM RPI-194. Representative number of experiments, *N* = 6 fibers or trabeculae for all measurements.

At baseline and in the absence of drug, the intrinsic calcium sensitivity of skeletal muscle fibers is about the same for limb slow (pCa_50_ 6.28 ± 0.03) and fast (6.27 ± 0.03) muscle fibers (see Figure 4 and Table 1). The calcium sensitivity of cardiac trabeculae is significantly lower (5.68 ± 0.02). Slow skeletal muscle has a higher calcium affinity than cardiac muscle, even though both muscle types utilize the same cTnC/ssTnC isoform.

**TABLE 1:** Fundamental properties of slow and fast fibers and of cardiac trabeculae, in the absence or presence of 20, 50 and 100 µM RPI-194.

	Slow Fibers	Fast Fibers	Cardiac Trabeculae
pCa_50_, series A, *N* = 24 of each	6.28 ± 0.03 (6.10–6.58)	6.27 ± 0.02 (6.03–6.46)	5.68 ± 0.01 (5.49–5.80)
∆pCa_50_ with 0 µM RPI-194 (series B), *N* = 6 of each	−0.01 ± 0.02 (−0.04–0.01)	0.04 ± 0.02 (−0.01–0.06)	0.00 ± 0.01 (−0.01–0.01)
∆pCa_50_ with 20 µM RPI-194 (series B), *N* = 6 of each	0.14 ± 0.02 (0.10–0.24)	0.04 ± 0.02 (−0.01–0.12)	0.03 ± 0.02 (−0.01–0.10)
∆pCa_50_ with 50 µM RPI-194 (series B), *N* = 6 of each	0.35 ± 0.02 (0.30–0.42)	0.14 ± 0.02 (0.07–0.19)	0.16 ± 0.02 (0.09–0.20)
∆pCa_50_ with 100 µM RPI-194 (series B), *N* = 6 of each	0.71 ± 0.05 (0.58–0.84)	0.25 ± 0.02 (0.14–0.27)	0.28 ± 0.02 (0.21–0.32)
V_o_ (FL/s), in the absence of RPI-194 (series A), *N* = 24 of each^a^	1.35 ± 0.04 (1.04–1.68)	4.53 ± 0.17 (3.19–6.31)	—
V_o_ ratio with 0 µM RPI-194 (series B/series A), *N* = 6 of each	0.94 ± 0.07 (0.83–1.02)	0.89 ± 0.04 (0.68–1.00)	—
V_o_ ratio with 20 µM RPI-194 (series B/series A), *N* = 6 of each	0.75 ± 0.03 (0.69–0.86)	0.67 ± 0.06 (0.51–0.82)	—
V_o_ ratio with 50 µM RPI-194 (series B/series A), *N* = 6 of each^a^	0.58 ± 0.04 (0.51–0.68)	0.61 ± 0.06 (0.49–0.85)	—
V_o_ ratio with 100 µM RPI-194 (series B/series A), *N* = 6 of each	0.50 ± 0.01 (0.48–0.52)	0.52 ± 0.04 (0.42–0.64)	—

a Values are mean ± SEM (range in parentheses). Here, V_o_ = maximal shortening velocity.

RPI-194 has a greater effect on slow skeletal muscle than cardiac muscle. The effects of RPI-194 were tested at 20, 50 and 100 µM concentrations. Six cardiac trabeculae, six slow fibers and six fast fibers were studied at each concentration, and each trabecula/fiber was used to study one concentration of the compound. The shift in the pCa_50_ (i.e., ∆pCa_50_) is significant between each tested concentration of RPI-194 within each muscle group. At 50 μM RPI-194, the pCa_50_ of slow fibers shifted +0.35 units, while the fast fibers and cardiac trabeculae shifted +0.14 and +0.16, respectively (Figure 4 and Table 1). Therefore, there is a larger calcium sensitizing effect of RPI-194 on limb slow fibers, compared to cardiac trabeculae and limb fast fibers. Although RPI-194 was designed and tested to bind to the cardiac/slow skeletal troponin complex, there is some cross-reactivity with fast skeletal muscle, but the effect is not nearly as large as that observed with tirasemtiv (+0.89) (Russell et al., 2012), which was designed specifically for fast skeletal muscle.

RPI-194 did not increase the maximum isometric force (P_o_) generated under saturating calcium concentrations in any of the muscle types (Supplementary Table S1). In fact, upon addition of 20, 50, or 100 μM RPI-194, there was an initial 15% decrease in P_o_ for the slow muscle fibers and a 10% decrease for fast muscle fibers and cardiac trabeculae, with no apparent concentration dependence at the concentrations tested. There was a trend towards recovery in P_o_ with time seen at higher concentrations of RPI-194 in cardiac trabeculae. The reasons behind these phenomena are not known. The skinned muscle fiber experiments are described in more detail in the Supplementary Material.

There was a marked effect of RPI-194 on maximal shortening velocity in unloaded slow and fast skeletal muscle fibers, with velocity generally decreasing with higher concentrations of RPI-194. The overall slowing effect of RPI-194 was similar in slow and fast fibers, with the velocity V_o_ being reduced to about half in the presence of 100 μM RPI-194 (Table 1).

Shortening velocity is determined by the load-dependent rate of actin-myosin cycling. It is possible that RPI-194 slows the velocity of unloaded contraction, V_0_, via a direct interaction with actin-myosin. We therefore proceeded to examine the effect of RPI-194 on human beta-cardiac myosin S1 ATPase activity in the presence of 40 µM actin. We found no effect whatsoever (see Supplementary Figure S2), suggesting that the reduction in V_0_ by RPI-194 seen in slow and fast skeletal muscle is not due to direct binding to actin-myosin S1, but rather, an effect on another myosin domain or light chain, troponin, or some other unknown off-target effect.

### RPI-194 Decreases Velocity and Amplitude of Contraction in Unloaded Individual Mouse Cardiomyocytes

**FIGURE 5:**
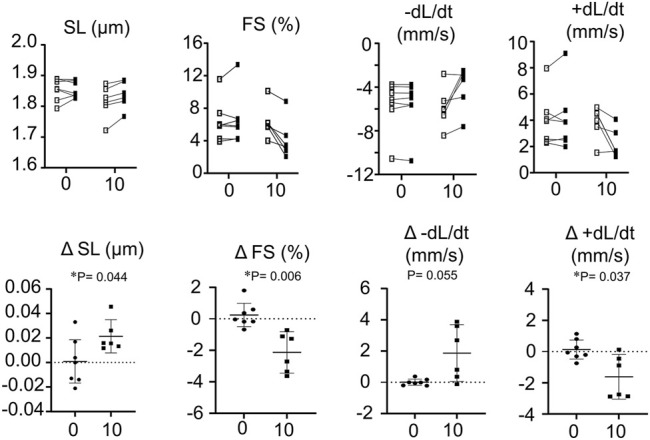
Top panel shows measurement of resting sarcomere length (SL), fractional shortening (FS), rate of contraction (-dL/dt) and rate of relaxation (+dL/dt) of isolated single cardiomyocytes. Open boxes represent baseline and filled boxes represent addition of either 0 or 10 µM RPI-194 (10). Bottom panel shows absolute changes of measurements from wild type values. *N* = 6 for all measurements. *p < 0.05 compared between placebo (0) or 10 µM RPI-194 (10) with Tukey post-hoc test.

The addition of RPI-194 to individual unloaded cardiomyocytes caused a decrease in observed contractility. 10 μM RPI-194 significantly increased resting sarcomere length, decreased fractional shortening, and decreased the velocity of contraction and relaxation (Figure 5). At a concentration of 100 μM, cardiomyocyte contractions ceased completely, with sarcomere lengths suggestive of a relaxed state (rather than a contracted state). The inhibition of contractility observed in cardiomyocytes contrasts with the increased calcium sensitivity of isometric contraction seen in skinned cardiac trabeculae. This raises the possibility that RPI-194 interferes with excitation-contraction coupling in living cells, for example, the inhibition of ion channels. When we attempted to measure calcium transients in cardiomyocytes using calcium-sensitive fluorophores, the strong intrinsic fluorescence of RPI-194 created too much background signal. It is therefore necessary to assess cardiac contractility in another system that includes intact cells.

### Cardiac Work is Maintained in Mouse Isolated Perfused Working Hearts

**FIGURE 6:**
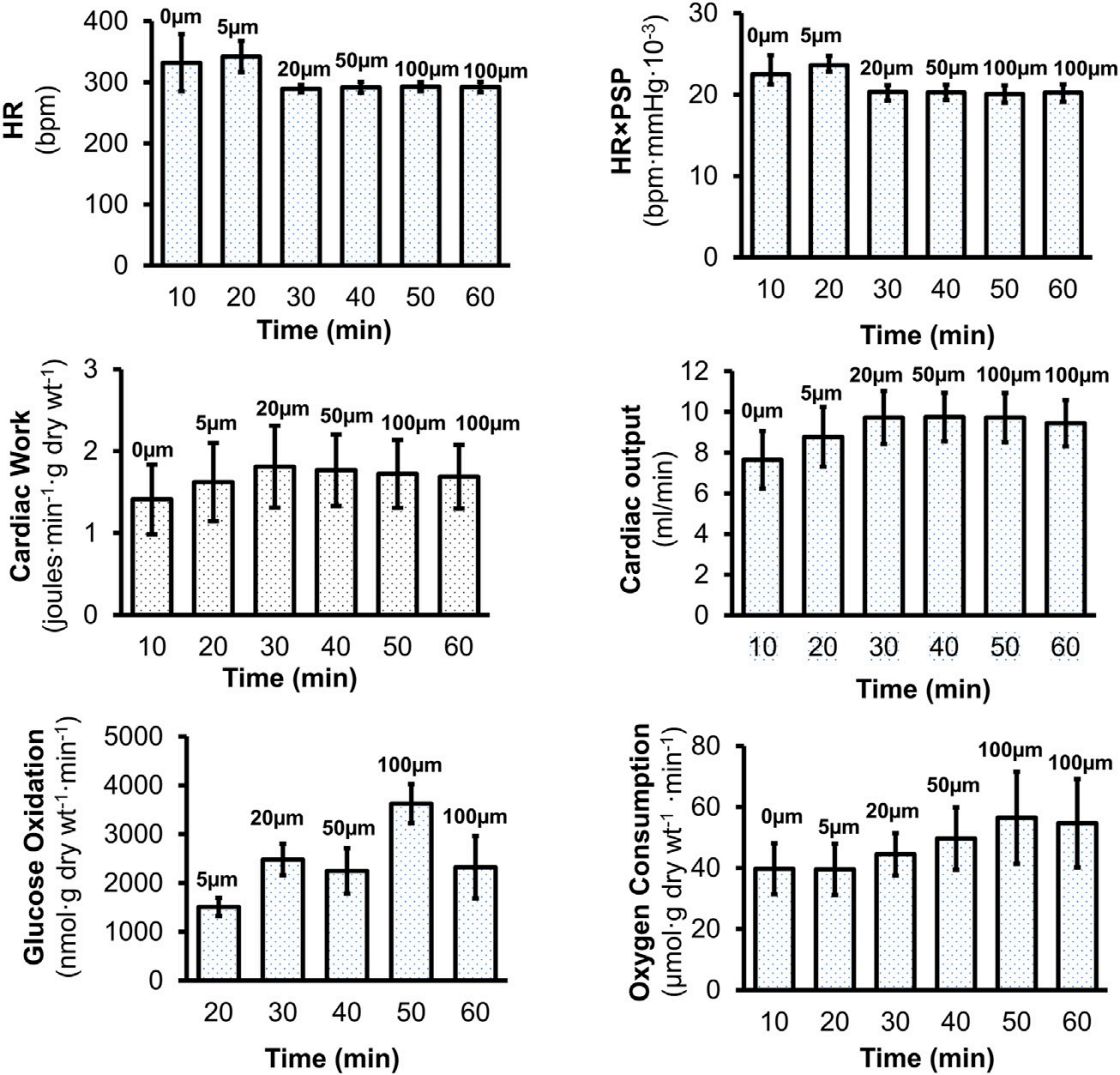
Effects of RPI-194 on heart rate (HR), heart rate×peak systolic pressure (HR×PSP), cardiac work, cardiac output, glucose oxidation, and oxygen consumption in isolated, perfused working mouse hearts. *N* = 4 for all measurements.

We studied the impact of RPI-194 in a mouse isolated perfused working heart model, which was maintained at constant pressure. There was no consistent trend in heart rate or heart rate times peak systolic pressure product as RPI-194 was added to the system up to a maximum of 100 µM (Figure 6). Cardiac output and cardiac work increased with increasing RPI-194 levels, but the trend was not statistically significant, and similar changes could also be observed in controls over the course of 60 min (see Supplementary Figure S3 **)**. As RPI-194 was added, the observed increase in cardiac work was accompanied by higher rates of glucose utilization and oxygen consumption. Thus, it appears that metabolic pathways are generally intact in the presence of RPI-194. The severe inhibitory effect of RPI-194 observed in individual unloaded cardiomyocytes was not observed in isolated perfused working hearts, making it less likely that the inhibitory effect observed in cardiomyocytes is due to modulation of ion channels.

## Discussion

Perhaps the most unexpected result in the current study is the effect of RPI-194 in slowing the velocity of unloaded shortening, both in skinned skeletal muscle fibers and in isolated cardiomyocytes. This could be due to an unknown off-target effect, although it is reassuring that cardiac function was preserved in mouse isolated perfused working hearts. It is possible that the decreased velocity of unloaded shortening seen with RPI-194 is a consequence of troponin activation itself. The ideal duty ratio (proportion of myosin heads strongly bound to actin) is dependent on load, with more myosin-actin interactions needed for higher loads, while for smaller loads excessive interactions might only contribute to drag. In isometric muscle contraction, actin-myosin interaction is maximal, with a duty ratio of about 0.25 (Land and Niederer, 2015). This value decreases to <0.05 in unloaded shortening, and it could be that excessive formation of actin-myosin cross-bridges beyond this lower duty ratio slows unloaded contraction (O’connell et al., 2007; Brizendine et al., 2015).

The effect of RPI-194 on unloaded cardiomyocytes is similar to that observed with mutations in cTnI or cTnT associated with hypertrophic cardiomyopathy (HCM) (Willott et al., 2010). Such mutations increase calcium sensitivity and shift the thin filament towards the activated state (Ren et al., 2018), much like the effect of RPI-194. Feline cardiomyocytes showed decreased amplitude and velocity of shortening after being transfected with R92Q-cTnT versus wildtype cTnT (Marian et al., 1997). A similar effect was observed in R92Q-cTnT transgenic mouse cardiomyocytes (Tardiff et al., 1999). Slowed contractility was also observed in isolated guinea pig cardiomyocytes transfected with R145G-cTnI. Thus, decreased and slowed contractions in unloaded cardiomyocytes appears to be a feature of HCM mutations, so it is possible that cardiac troponin activation by RPI-194 has a similar effect. Nevertheless, an off-target effect remains a possibility.

RPI-194 was designed to bind and stabilize the calcium-bound activated complex between cardiac TnC and TnI. The structure of RPI-194 can be further engineered to improve its specificity by adding aromatic ring substituents or restricting its degrees of freedom. This would help to resolve whether the observed slowing of unloaded shortening seen with RPI-194 is due to its impact on troponin or an off-target effect.

It is unlikely that any modifications of RPI-194 would enhance its specificity for cardiac troponin with respect to slow skeletal muscle troponin. Both use the same troponin C isoform, and the residues in the switch region binding the RPI-194 molecule are highly homologous in the troponin I isoforms (compare Ile148 and Met153, cTnI, in Figure 1A *versus* Val118 and Met123, ssTnI, in Figure 2A). It is possible that RPI-194 could be modified to decrease cross-reactivity with fast skeletal troponin, for which there are selective activators, tirasemtiv and reldesemtiv (Russell et al., 2012; Hwee et al., 2014; Hwee et al., 2015; Calder et al., 2016). Whether or not cross-reactivity with skeletal muscle, particularly slow skeletal muscle, would limit potential use of a cardiac troponin activator, remains to be seen. On the other hand, it is also unknown whether the cardiac effects of a general troponin activator would limit its use as slow skeletal muscle activator. Whole animal models examining the impact of compounds like RPI-194 are needed.

Finally, we note that our cardiac troponin activator RPI-194 likely has a different mechanism of activity from the recently published cardiac troponin activator TA1, a closely related analog of the drug AMG-594/CK-136, which has undergone Phase 1 clinical trials (https://cytokinetics.com/ck-136/). TA1/AMG-594/CK-136 is highly selective for cardiac muscle over slow skeletal muscle, which would not be possible if it were targeting the same binding site as RPI-194. Moreover, TA1 is more potent, not only causing a greater leftward shift for pCa_50_ in cardiac trabeculae, but also markedly increasing the maximum force generated at saturating calcium concentrations, unlike RPI-194 (He et al., 2021). Increased force was also observed at resting calcium concentrations, along with increased myosin ATP consumption in cardiac myofibrils. The behaviour of RPI-194 is more in keeping with what was observed for fast skeletal troponin activator tirasemtiv, with a leftward pCa_50_ but no significant change in force generated at low or saturating calcium concentrations (Russell et al., 2012), which is not surprising given that they both target the same homologous binding pocket (Li et al., 2021). It is possible that TA1 is able to activate the thin filament through cTnI/cTnT, independent of the calcium binding activity of cTnC, unlike RPI-194 and tirasemtiv. Further comparative studies are needed to delineate the differences between RPI-194 and TA1/AMG-594/CK-136 in terms of mechanism of action and physiologic impact.

## Data Availability

The original contributions presented in the study are included in the article/Supplementary Material, further inquiries can be directed to the corresponding authors.

## Supplementary Material

Supplementary Figure S1 Fluorescence signal of 5 µM RPI-194 with titration of gChimera protein (left), or titration of cNTnC protein (right). *N* = 3 for all measurements.

Supplementary Figure S2 Lack of impact of RPI-194 on myosin ATPase activity. The ATPase activity of human beta-cardiac myosin subfragment 1 (0.1 µM) was measured in the presence of 40 µM actin and varying concentrations of drug (RPI-194P). The ATPase activity is reported as moles of Pi per mole of myosin per second. Number of experiments with separate protein preparations, *N* = 3.

Supplementary Figure S3 Effects of DMSO in heart rate (HR), heart rate×peak systolic pressure (HR×PSP), cardiac work, and cardiac output in isolated, perfused working mouse hearts. N = 7 for all measurements.
