# Supplementary material for: Small Molecule RPI-194 Stabilizes Activated Troponin to Increase the Calcium Sensitivity of Striated Muscle Contraction
Source: Front Physiol. 2022 Jun 8;13:892979. doi: 10.3389/fphys.2022.892979 (PMC9213791; doi:10.3389/fphys.2022.892979)
Supplement: Supplementary file 1 [file Table1.DOCX]

**Supplementary table 1**: Fundamental properties of slow and fast fibers and of cardiac trabeculae, in the absence or presence of 20, 50 and 100 µM RPI-194. Values are mean ± SEM (range in parentheses). Here, P_o_= maximum isometric force, CSA= cross-sectional area, SL= sarcomere length, and FL= fiber length.

|  | Slow Fibers | Fast Fibers | Cardiac Trabeculae |
| --- | --- | --- | --- |
| Storage duration (days),  N = 24 of each | 14 ± 1  (1-26) | 12 ± 1  (1-24) | 10 ± 2  (1-27) |
|  |  |  |  |
| Cross-sectional area (µm^2^),  N = 24 of each | 6,901 ± 284  (4,885-10,282) | 10,441 ± 577  (5.756-18,271) | 37,284 ± 2,957  (8,651-71,409) |
|  |  |  |  |
| Resting SL (um),  N = 24 of each | 2.47 ± 0.01  (2.39-2.57) | 2.47 ± 0.01  (2.41-2.55) | 2.07 ± 0.02  (1.93-2.19) |
|  |  |  |  |
| P_o_/CSA (kN/m^2^,  N = 24 of each | 104.5 ± 3.2  (66.6-121.1) | 91.6 ± 4.1  (56.0-118.7) | 14.6 ± 0.9  (6.4-24.1) |
|  |  |  |  |
| Last P_o_ in series A/first P_o_, in series A,  N = 24 of each | 0.98 ± 0.01  (0.81-1.11) | 1.05 ± 0.01  (0.89-1.17) | 0.92 ± 0.02  (0.83-1.10) |
|  |  |  |  |
| First P_o_ with 0 µM RPI-194 in series B/last P_o_ in series A,  N = 6 of each | 0.99 ± 0.01  (0.97-1.01) | 0.98 ± 0.02  (0.92-1.02) | 0.96 ± 0.01  (0.93-1.00) |
|  |  |  |  |
| First P_o_ with 20 µM RPI-194 in series B/last P_o_ in series A,  N = 6 of each | 0.85 ± 0.02  (0.81-0.91) | 0.91 ± 0.01  (0.87-0.95) | 0.89 ± 0.01  (0.84-0.93) |
|  |  |  |  |
| First P_o_ with 50 µM RPI-194 in series B/last P_o_ in series A,  N = 6 of each | 0.84 ± 0.02  (0.81-0.91) | 0.92 ± 0.06  (0.84-1.03) | 0.87 ± 0.03  (0.81-0.98) |
|  |  |  |  |
| First P_o_ with 100 µM RPI-194 in series B/last P_o_ in series A,  N = 6 of each | 0.85 ± 0.02  (0.78-0.92) | 0.90 ± 0.01  (0.85-0.94) | 0.92 ± 0.02  (0.86-1.00) |
|  |  |  |  |
| Last P_o_ with 0 µM RPI-194 in series B/First P_o_ in series B,  N = 6 of each | 1.00 ± 0.01  (0.97-1.04) | 1.04 ± 0.03  (0.98-1.08 | 0.98 ± 0.02  (0.97-1.04) |
|  |  |  |  |
| Last P_o_ with 20 µM RPI-194 in series B/First P_o_ in series B,  N = 6 of each | 1.02 ± 0.01  (0.97-1.05) | 1.03 ± 0.02  (0.95-1.07) | 0.97 ± 0.03  (0.86-1.07) |
|  |  |  |  |
| Last P_o_ with 50 µM RPI-194/in series B/First P_o_ in series B,  N = 6 of each | 1.03 ± 0.02  (0.96-1.07) | 1.06 ± 0.02  (0.99-1.12) | 1.01 ± 0.04  (0.84-1.09) |
|  |  |  |  |
| Last P_o_ with 100 µM RPI-194 in series B/First P_o_ in series B, N = 6 of each | 1.04 ± 0.02  (0.95-1.09) | - 1. ±0.02   (0.97-1.07) | 1.11 ± 0.02  (1.04-1.18) |
