# Supplementary figures and images for: Small Molecule RPI-194 Stabilizes Activated Troponin to Increase the Calcium Sensitivity of Striated Muscle Contraction
Source: Front Physiol. 2022 Jun 8;13:892979. doi: 10.3389/fphys.2022.892979 (PMC9213791; doi:10.3389/fphys.2022.892979)

## Supplementary figure S2

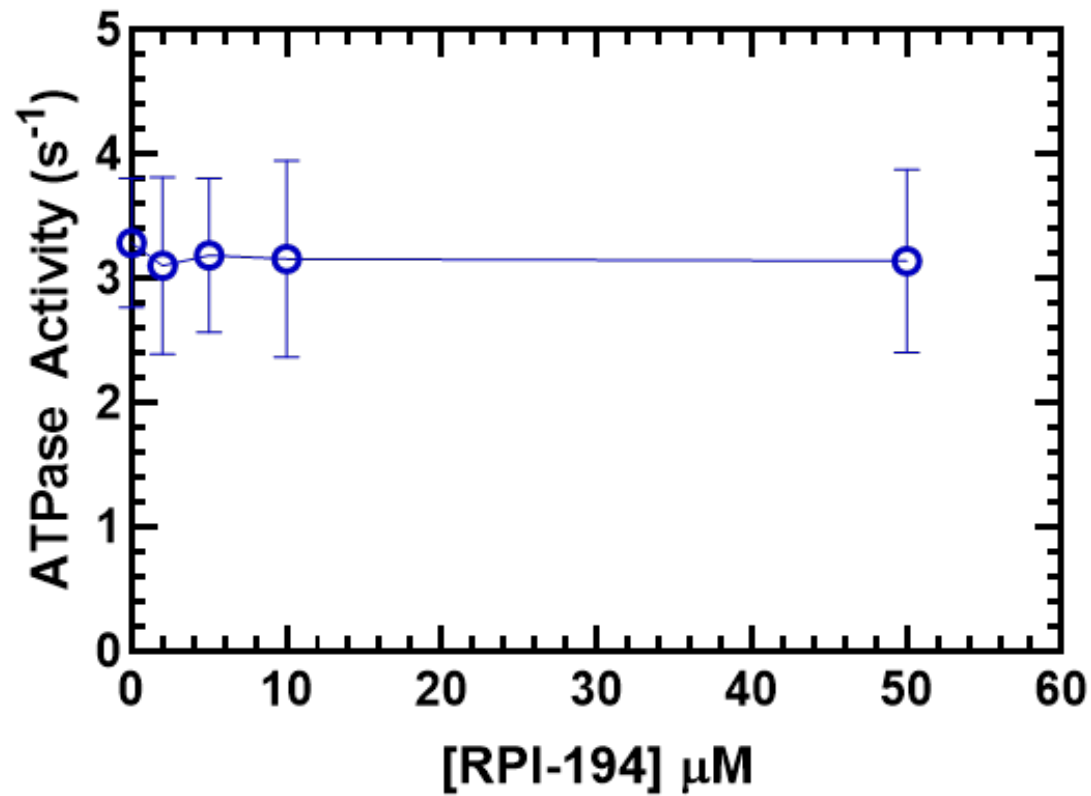

Supplement: Supplementary file 2 [file Image2.pdf]

Supplementary figure S3

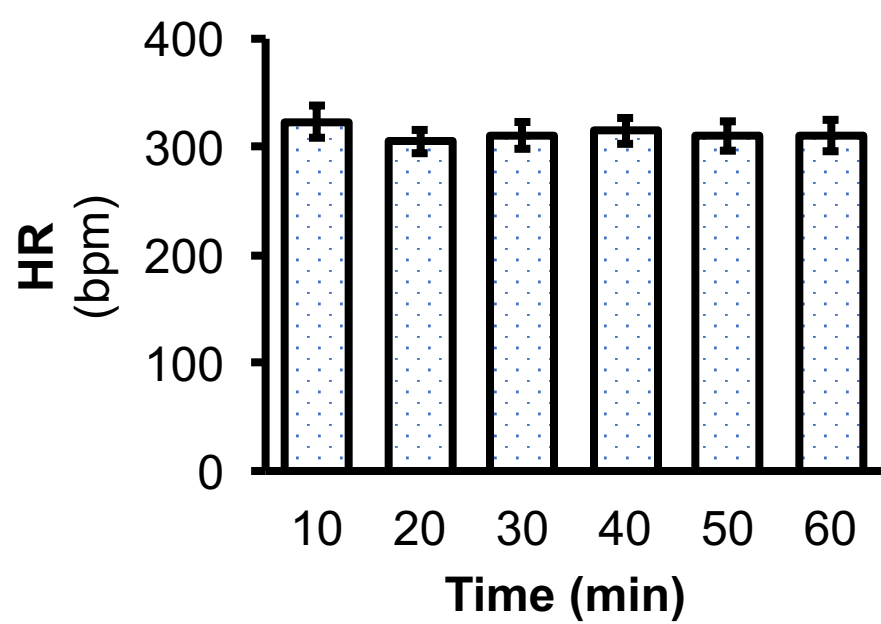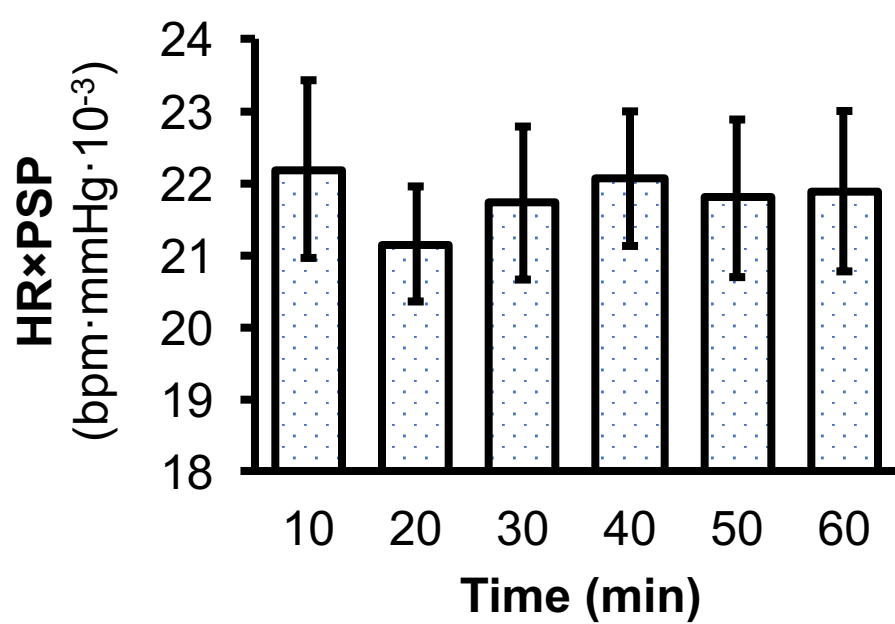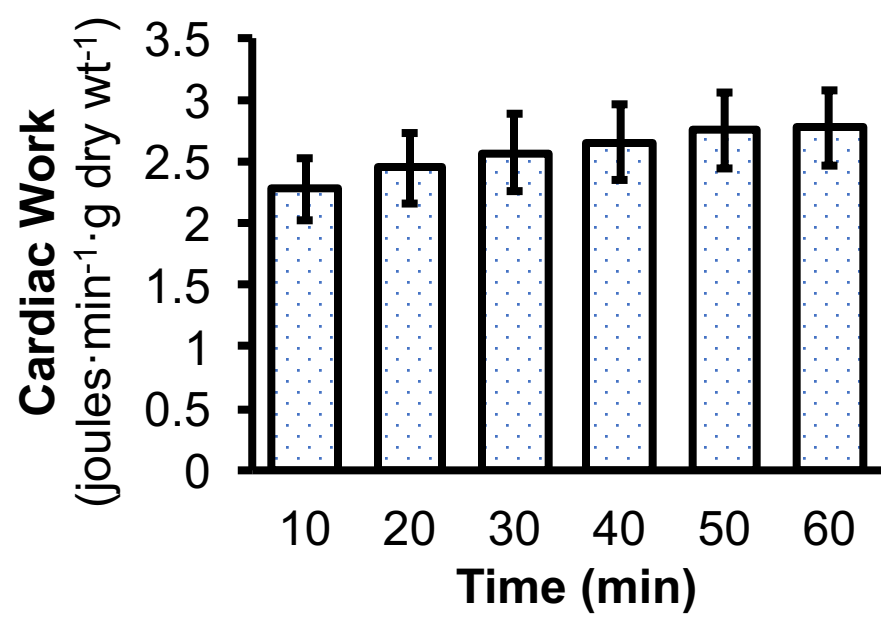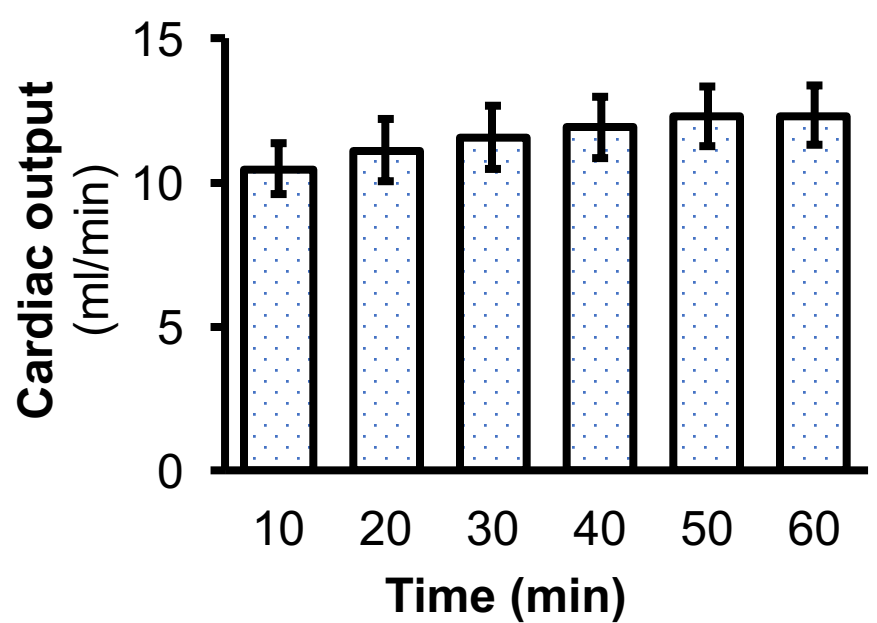

Supplement: Supplementary file 3 [file Image3.pdf]

# Supplementary figure S1

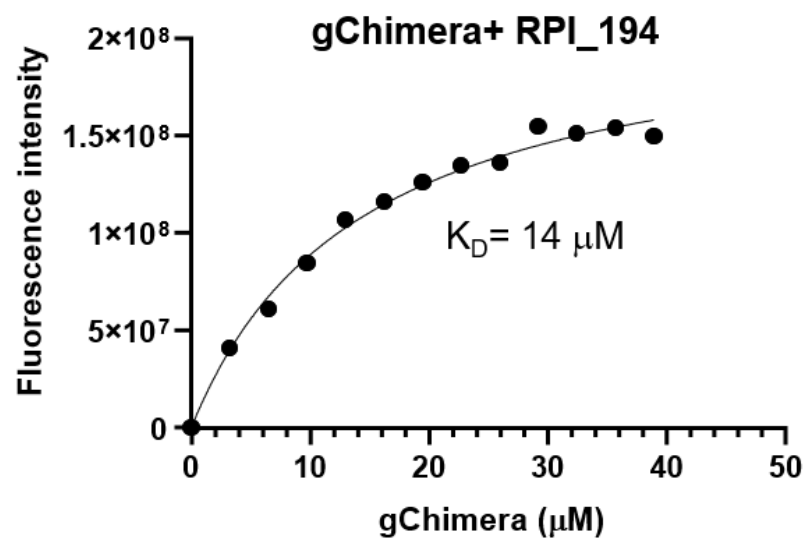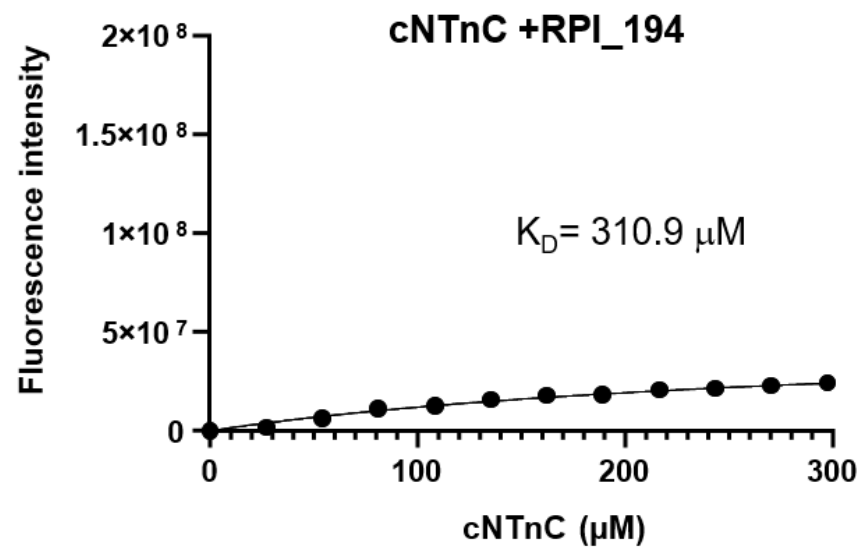

Supplement: Supplementary file 5 [file Image1.pdf]
