# Supplementary material for: Small Molecule RPI-194 Stabilizes Activated Troponin to Increase the Calcium Sensitivity of Striated Muscle Contraction
Source: Front Physiol. 2022 Jun 8;13:892979. doi: 10.3389/fphys.2022.892979 (PMC9213791; doi:10.3389/fphys.2022.892979)
Supplement: Supplementary file 4 [file DataSheet1.docx]

**Small molecule RPI-194 stabilizes activated troponin to increase the calcium sensitivity of striated muscle contraction**

Zabed Mahmud^1^, Svetlana Tikunova^2^, Natalya Belevych^3^, Cory S. Wagg^4^, Pavel Zhabyeyev^5^, Philip B. Liu^5^, David V. Rasicci^6^, Christopher M. Yengo^6^, Gavin Y. Oudit^5^, Gary D. Lopaschuk^4^, Peter J. Reiser^3,*^, Jonathan P. Davis^2,*^, and Peter M. Hwang^1,5*^

**Affiliations:**

1 Department of Biochemistry, University of Alberta, Edmonton, AB, Canada

2 Department of Physiology and Cell Biology, The Ohio State University, Columbus, OH, USA

3 Division of Biosciences, College of Dentistry, The Ohio State University, Columbus, OH, USA

4 Department of Pediatrics, University of Alberta, Edmonton, AB, Canada

5 Department of Medicine, University of Alberta, Edmonton, AB, Canada

6 Department of Cellular and Molecular Physiology, College of Medicine, Pennsylvania State University, Hershey, PA, USA

^*^To whom correspondence should be addressed:

Corresponding authors**:**

Peter M. Hwang

Email: [phwang1@ualberta.ca](mailto:phwang1@ualberta.ca)

Peter J. Reiser

Email: [reiser.17@osu.edu](mailto:reiser.17@osu.edu)

Jonathan P. Davis

Email: [davis.812@osu.edu](mailto:davis.812@osu.edu)

**Keywords:**

RPI-194, cardiac troponin activator, calcium sensitizer, inotrope, systolic heart failure, striated muscle, thin filament

**RPI-194 activates cardiac, slow skeletal, and fast skeletal muscle in isometric contraction, but slows the velocity of unloaded contraction**

The mean ± SEM of all of the parameters that were measured, first without (series A) and then with 20, 50 and 100 µM RPI-194 (series B), are presented in Table 1. The mean CSA of slow and fast fibers was 7,114 µm^2^ and 10,676 µm^2^, respectively. The mean size of the cardiac trabeculae was 40,656 um^2^. The mean resting sarcomere length (SL) was 2.48 um in slow fibers and 2.47 in fast fibers. The variation in the inherent calcium sensitivity (pCa_50_ without RPI-194, series A) was not related to the variation in resting SL in either slow or fast fibers (linear regression, R^2^ = 0.0476 and 0.0129, respectively). The mean resting SL in cardiac trabeculae was 2.06 um and the variation in the inherent calcium sensitivity (series A) was not related to the variation in resting SL (R^2^ = 0.1669; least squares linear regression, P = 0.092).

The mean duration of storage of slow fibers, fast fibers and cardiac trabeculae, at -20^o^C prior to force/pCa measurements, was 13, 12 and 9 days, respectively. The variation in the inherent calcium sensitivity was not related to the variation in storage duration for fast or slow fibers or for trabeculae (least squares linear regression, P = 0.173, 0.615 and 0.083, respectively; SYSTAT).

The greatest force (P_o_) generated in pCa 4.0 during the series of force/pCa measurements without RPI-194 (series A) in each fiber/trabecula was normalized with cross-sectional area (CSA; not corrected for swelling consequent to skinning) (Godt and Maughan, 1977). The mean P_o_/CSA (kN/m^2^) in slow and fast fibers was 104.5 and 91.6, respectively, and was 14.3 in trabeculae. The variation in P_o_/CSA in slow and fast fibers and in trabeculae was not related to storage duration (least square linear regression, P = 0.104, 0.116 and 0.425, respectively). The variation in the inherent calcium sensitivity was not related to the variation in P_o_/CSA in slow fibers or in trabeculae (least square linear regression, P= 0.959 and 0.202 for slow fibers and trabeculae, respectively, SYSTAT), but was correlated in fast fibers (R^2^ = 0.277, P = 0.025), with fast fibers that generated lower P_o_/CSA having lower calcium sensitivity. This could suggest that fibers that were stored longer and had lower P_o_/CSA partially lost troponin C during greater storage duration, but these two parameters (i.e., storage duration and calcium sensitivity) were not correlated in slow or fast limb fibers or in trabeculae. This suggests that fast fibers with higher force/area are generally more calcium sensitive, which could augment power production by generating greater force (Power = Force X Velocity), but the basis for stronger fast fibers having greater calcium sensitivity was not probed.

The first and last activations, of each fiber or trabecula, for all of the measurements of calcium sensitivity (with or without RPI-194) were in pCa 4.0 solution (maximally activating). To assess the stability of the maximal force generating ability of each preparation during measurements, the ratio of the force during the last activation to the force during the first activation without RPI-194 (series A) was calculated. The average ratio was 0.98 in slow fibers, 1.05 in fast fibers and 0.92 in trabeculae. The average value of the same ratio, in the presence of RPI-194 (series B), was ~1.03, independent of the concentration of RPI-194 in slow and fast fibers. The average value of this ratio increased significantly from 20 to 100 µM RPI-194 in trabeculae and was significantly greater with 100 µM RPI-194 in trabeculae, compared to slow and fast fibers.

**Baseline properties of fast and slow fibers and of trabeculae, in the absence of RPI-194:**

In the absence of RPI-194, there was no difference in pCa_50_ between fast and slow fibers but pCa_50_ in trabeculae differed significantly from that in fast and slow fibers. The mean ± SEM differences between the first (series A) and second (series B) pCa_50_ values in the slow and fast fibers and trabeculae, in the absence of RPI-194, were -0.01 ± 0.01, 0.04 ± 0.02, and 0.00 ± 0.00, respectively. None of these values were statistically significant. The mean ± SEM ratio of the series B V_o_ determination/series A V_o_ determination in the slow and fast fibers was 0.94 ± 0.03 and 0.89 ± 0.04, respectively. The second V_o_ values were not significantly different from the first V_o_ in either slow or fast fibers. Therefore, the pCa_50_ and V_o_ were essentially the same when measured twice in a given preparation without RPI-194.

**Statistically significant results with RPI-194 in fast fibers:**

pCa_50_ was shifted by 50 and 100 µM RPI-194, but not be 20 µM. The shift was greater with 100 µM than with 50 µM and both of these concentrations caused a greater shift than with 20 µM.

The force generated during the first maximal activation (at pCa 4.0) in 20, 50 and 100 µM RPI-194, in series B, was reduced, on average, by 10%, compared to the force generated during the final maximal activation, in series A (without RPI-194). There was a mean 3% recovery in maximal force generation across all twenty-four fast fibers, by the end of the measurements in series B, independent of RPI-194 concentration.

V_o_ decreased with 20, 50 and 100 µM RPI-194, compared to 0 µM, but it did not differ across these concentrations.

The Pearson correlation coefficient between the V_o_ ratio and the shift in pCa_50_ across all three concentrations of RPI-194 was -0.410 and was not statistically significant (P = 0.102).

**Statistically significant results with RPI-194 in trabeculae:**

The RPI-194- induced shifts in pCa_50_ in trabeculae, had the same concentration dependency as in fast fibers. There was no shift with 20 µM RPI-194, a shift with 50 µM, and a greater shift with 100 µM.

The maximal force generated during the first activation (at pCa 4.0) in 20, 50 and 100 µM RPI-194, in series B, was reduced, on average, by 11%, compared to the force generated during the final maximal activation, in series A (without RPI-194). There was no significant subsequent change in maximal force generation during series B, except with 100 µM during which maximal force increased on average by 11%.

**Statistically significant results in with RPI-194 slow fibers:**

pCa_50_ was shifted by 20, 50 and 100 µM RPI-194 and the shift was dose-dependent across all three concentrations. The shift in pCa_50_ was greater in slow fibers than in fast fibers and in trabeculae at each concentration of RPI-194.

The force generated during the first maximal activation (at pCa 4.0) in 20, 50 and 100 µM RPI-194, in series B, was reduced by 15%, 16% and 15%, respectively, compared to the force generated during the final maximal activation, in series A (without RPI-194). There was a mean 2% recovery in maximal force generation across all twenty-four slow fibers, by the end of the measurements in series B, independent of RPI-194 concentration.

V_o_ decreased with 20, 50 and 100 µM RPI-194, compared to 0 µM RPI-194 and the decrease was dose-dependent up to 50 µM.

The Pearson correlation coefficient between the V_o_ ratio and the shift in pCa_50_ across all three concentrations of RPI-194 was -0.798, indicating a strong relationship (P < 0.00001). The coefficient of determination was 0.6368.

**Comparison of RPI-194-shifts in calcium sensitivity in slow and fast fibers and trabeculae:**

The shift in the pCa_50_ at each concentration of RPI-194 was significantly different between slow fibers and fast fibers and between slow fibers and trabeculae and was not different at any of the tested concentrations, between fast fibers and trabeculae. Therefore, limb slow fibers are markedly more sensitive to RPI-194, compared to cardiac trabeculae and limb fast fibers.

**Reference:**

Godt, R.E., and Maughan, D.W. (1977). Swelling of skinned muscle fibers of the frog. Experimental observations. *Biophys J* 19**,** 103-116. doi: 10.1016/S0006-3495(77)85573-2.
